# Does spasticity affect the postural stability and quality of life of children with cerebral palsy?

**DOI:** 10.1016/j.jtumed.2021.04.011

**Published:** 2021-06-11

**Authors:** Mostafa S. Ali

**Affiliations:** Department of Pediatrics, Faculty of Physical Therapy, Cairo University, Egypt

**Keywords:** الشلل الدماغي, الوظيفة, التشنج, الاستقرار الوضعي, نوعية الحياة, Cerebral palsy, Function, Postural stability, Quality of life, Spasticity

## Abstract

**Objectives:**

Cerebral palsy is a unique physical disability that primarily affects children's gross motor functions and postural control. Cerebral palsy has a direct impact on children's daily activities and quality of life. This study aims to determine the relationship between spasticity, motor function, postural stability, and the quality life of children with cerebral palsy.

**Methods:**

Forty-five children (age range 4–6 years) diagnosed with spasticity from cerebral palsy participated in this study. Spasticity was evaluated by the modified Ashwarth scale; the children's functions were evaluated by gross motor functional measures, postural stability was evaluated by biodex balance system, and quality of life was measured with the pediatric quality of life inventory.

**Results:**

There was a strong positive correlation between the degree of spasticity and quality of life. Additionally, there was a significantly strong association between spasticity and gross motor function. In contrast, there was no correlation between spasticity and postural stability indices. Moreover, there was a strong positive correlation between quality of life and gross motor function. Lastly, there was no association between quality of life and the postural stability index.

**Conclusion:**

The findings highlight the impact of spasticity on motor function and the quality of life of a cohort of children with cerebral palsy. These findings may determine therapeutic interventions and priorities to plan physical therapy programs. Such measures may overcome the main cause of disorders that delay and undermine the daily routines of the affected children.

## **الملخص**

**أهداف البحث:**

الشلل الدماغي هو إعاقة جسدية فريدة تؤثر في الغالب على الوظائف الحركية الكبرى للأطفال مع تأثير مباشر على التحكم في الوضع. للشلل الدماغي تأثير مباشر على نشاط الطفل اليومي مع تأثيره على نوعية الحياة. تهدف هذه الدراسة إلى تحديد العلاقة بين التشنج، والوظيفة الحركية، والاستقرار الوضعي، ونوعية الحياة لدى الأطفال المصابين بالشلل الدماغي.

**طرق البحث:**

شارك في هذه الدراسة ٤٥ طفلا (تتراوح أعمارهم من ٤ إلى ٦ سنوات)، تم تشخيص إصابتهم بالتشنج الناتج عن الشلل الدماغي. وتم تقييم التشنج بمقياس أشوارث المعدل. كما تم تقييم وظيفة الطفل من خلال المقاييس الوظيفية الحركية الإجمالية، وتم تقييم ثبات الوضع من خلال نظام توازن بيودكس، في حين تم قياس جودة الحياة من خلال موجودات جودة حياة الأطفال.

**النتائج:**

توجد علاقة ارتباط موجبة قوية بين درجة التشنج ونسبة جودة الحياة. بالإضافة إلى ذلك، كان هناك ارتباط قوي بشكل ملحوظ بين التشنج والوظيفة الحركية الإجمالية. في المقابل، لم يكن هناك ارتباط بين مؤشرات استقرار التشنج والوضعية. علاوة على ذلك، كان هناك ارتباط إيجابي قوي بين نوعية الحياة والوظيفة الحركية الإجمالية. أخيرا، لم يكن هناك ارتباط بين النسبة المئوية لجودة الحياة ومؤشر ثبات الوضع.

**الاستنتاجات:**

تسلط النتائج الضوء على تأثير التشنج على الوظيفة الحركية ونسبة صحة الجودة في مجموعة من الأطفال المصابين بالشلل الدماغي. قد تحدد هذه النتائج التدخلات العلاجية والأولويات لتخطيط برامج العلاج الطبيعي. وقد تتغلب هذه الإجراءات على السبب الرئيس للاضطراب الذي يؤخر ويقوض الروتين اليومي للأطفال المصابين.

## Introduction

Cerebral palsy (CP) is the primary origin of physical incapacity in children, with an expected frequency of 2.11 per 1,000 live births.^1^ Impaired development of movement and posture are essential for CP diagnoses.^2^ The problems associated with movement and posture in children with CP include abnormal muscle tone and spasticity, activity limitation, lack of equilibrium and alterations in the alignment that affect motor development, and gross motor function.^2^

Children with spastic CP are characterized by increased muscle tone, paresis, and involuntary motor control, and usually have difficulty in maintaining balance in an upright posture, which require a high center of mass and small base of support.^3^ Spasticity is a concern and involves neuromuscular syndromes that affect the health life quality of children with CP.^4^

Spasticity can lead to functional disorders associated with children's daily activities such as walking, dressing, eating, going to the toilet, and hygiene. Furthermore, spasticity can cause pain; muscular spasms; limitations of movement in bed; improper transfers; delayed development in sitting, standing, and walking; abnormal posture; and joint and bone deformity as a result of shorting and contractures that may lead to subluxation or dislocation with the children finally becoming full dependent.^5^

Postural and movement disorders that result in muscle tone abnormality, functional restriction, and improper body equilibrium and righting can affect sitting posture and lead to compensatory positions in the cardinal planes.^6^^,^^7^ This can result in a diminished ability to preserve one's posture against gravity due to inadequate trunk control and delayed limb movement. Abnormal posture and delayed development of motor control resulting in delayed sitting and standing are the main causes of postural instability.^8^

Postural stability is the ability to maintain the position of one's body in space to maintain alignment and control.^9^^,^^10^ Balance or postural control can be defined as the ability to preserve the center of body mass within its control area.^9^^,^^11^ Steady sitting positions for children with CP can maximize their development of hand-eye coordination, upper extremities functions, self-care and skill functions, cognitive development, and social communication.^12^^,^^13^

Health-related quality of life (HRQoL) is an individual multidimensional concept that measures the physical, social, and psychological aspects of children's health.^14^ HRQoL is an essential tool for assessing children and adolescents with CP.^15^

The functional deficits of children with CP due to the associated impairments of physical activities, social difficulties, cognitive delay, sensory disorders, and emotional problems lead to reductions in children's ability to accomplish their allocated-social communications and major breakdowns in HRQOL levels.^16^^,^^17^

QOL is an applicable health indicator that provides evidence about children's clinical state that influences their life and guides public policies for improving QOL.^18^ The National Policy on Health Promotion (PNPS) highlights the hunt for value and proposes the advancement of QOL and the decline of health threats related to conditioning variables, including lifestyle, education, culture, environment, and fundamental services.^19^

This study attempts to understand whether spasticity affects motor function, postural stability, and health life quality. Thus, the purpose of current study was to determine the relationship between spasticity, function, postural stability, and QOL in children with CP.

## Materials and Methods

This study was conducted between April and October 2020. Parents were asked to an informed consent forms and agree to the participation and evaluation of their children in the study. Forty-five children with spastic CP were selected from the outpatient clinic of faculty of physical therapy at Cairo University. The inclusion criteria were a) spastic CP, b) age of 4–6 years of both genders, and c) spasticity grades 1 and 1+ on the modified Ashworth scale,^20^ while the exclusion criteria were a) permanent deformities or severe contractures of upper or lower limbs or vertebral column, b) breathing disorders, c) an epilepsy or seizure disorder that was resistant to treatment, or d) orthopedic surgery owing to pathology and Botox injections during the 12 months that preceded the study.

### Procedures

1. **The modified Ashworth scale** is quick and easy valid test used to grade the degree of spasticity that does not require any equipment. It is performed manually to determine the resistance of lower limb muscles to passive stretching as a measure of degree of spasticity.^20^ All selected children were affected by spasticity classified with grades 1 and 1+.^21^
2. **Gross Motor Functional Measurement-88 (GMFM-88):** is a standard principle-test aiming to evaluate alterations of function in different milestones for children diagnosed with CP.^22^ There were 88 total items divided into five scopes: a) lying and rolling, b) sitting, c) crawling and kneeling, d) standing e) walking, running, and jumping. A 4-point Likert scale was used with the score. The GMFM scores are acceptably reliable for children with CP.^23^ The GMFCS has been successfully implemented worldwide in a range of settings including routine clinical management.^20^
3. **The pediatric quality of life inventory**^**“TM”**^**(PedsQL**^**TM**^**):** Health-related QOL was used to assess QOL by (PedsQL™) 4.0 generic core scale which measures HRQOL in normal children and adolescents as well as those with acute or chronic disorders. PedsQL™ is a reliable and valid measurement of the pediatric health outcome, as it enables the evaluation of risk.^24^ The Arabic version of PedsQL™ 4.0 created by Arabiat et al.^25^ reflected adequate psychometric properties. The PedsQL™ 4.0 is suitable for children of all ages. In children older than 4 (5–7, 8–12, and 13–18 years), it includes parallel child self-reports and parent proxy reports and is composed of 23 items. Furthermore, in children between 2 and 4 years, a parent proxy report was used with 21 items. The scale was applied and analyzed according to PedsQL™ guidelines. The selected children were asked to identify problems related to physical, social, and emotional functions and school performance (total 23 items) that had occurred during the study. The items were scored on a 5-point Likert scale, with score 0 indicating no problem, score 1 indicating almost no problem, score 2 indicating occasional problems, score 3 indicating frequent problems, and score 4 indicating almost constant problems, with a final total score value of 100, 75, 50, 25, or 0. A higher score indicates a greater QOL, superior health, or a better function. The PedsQL revealed better reliability and validity for children with acute or chronic wellbeing problems, along with healthy children and teenagers.^26^
4. **Biodex balance system** was used to evaluate the postural stability and dynamic balance of children through the tilting of a platform. The system was used to assess all stability indices. All instructions of test performance were explained to the children before applying the test. Children were then asked to stand in the middle of the platform with both legs and to look at a screen for feedback after adjusting the handrails to achieve the optimal safety. Then, children were instructed to stabilize the upright position on the middle of platform, as imitated in the mid screen feedback area, to stabilize the platform and determine foot angles. After introducing the angles to the device, the test starts, and the child is asked to look at the screen and maintain the cursor in the middle while the platform moves. Finally, a report is printed with stability indices.

### Sample size

**Figure 1:**
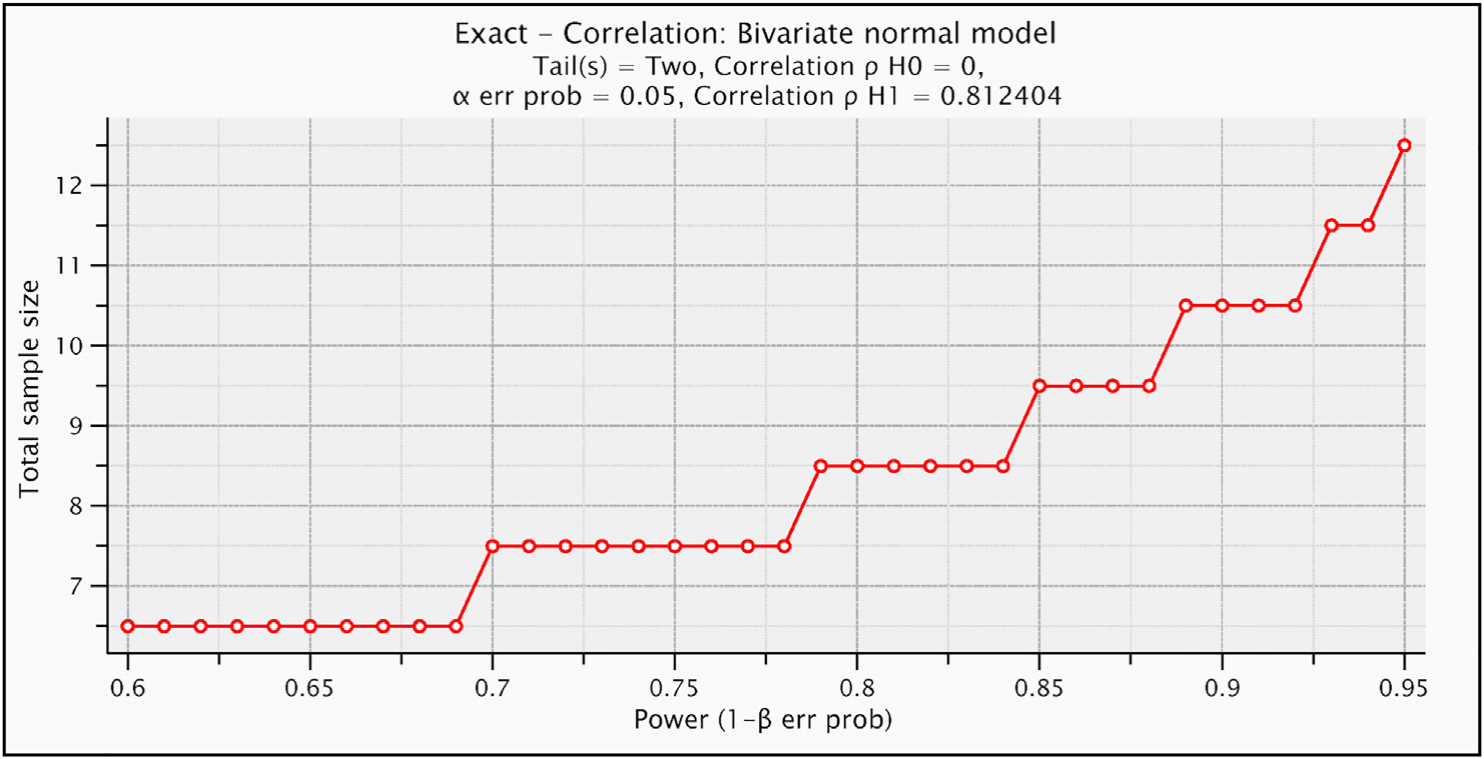
Plot of sample size calculation.

G∗POWER statistical programming was used to test size estimation [Exact- Correlation: Bivariate normal model, α = 0.05, β = 0.20, number of variables = 2, correlation **ρH0** = 0, correlation **ρH1** = 0.81, and coefficient of determination **ρ**^**2**^ = 0.66 and shown that the suitable sample size of current study was N = 13]. This coefficient of determination **ρ**^**2**^ was calculated from a pilot study of ten participants (between grades of spasticity and gross motor function) (Figure 1).

### Data analysis

Data analysis was conducted using SPSS for windows, version 23 (SPSS, Inc., Chicago, IL). The Spearman product–moment correlation was used to determine the strength and direction of the linear relationship between the ordinal variable (spasticity grades) and continuous variables (pedsQL, GMFM, 3 stability indices). While Pearson product–moment correlation was used to determine the strength and direction of the linear relationship between the continuous variable (Ped QL) and continuous variables (pedsQL, GMFM, 3 stability indices). The alpha level was set at 0.05.

## Results

Forty-five participants (22 girls, 23 boys) were included in the current study. Their ages ranged between 4 and 6 years with mean 4.23 ± 0.98. The descriptive statistics of the study are expressed in Table 1. The Spearman's rank-order correlation between spasticity grades and pedsQl revealed a positive strong correlation (p < 0.05). This means that an increase in the spasticity grades is consistent with an increase in PedsQL. Further, there was a positive strong correlation (p < 0.05) between spasticity grades and gross motor function. This means that an increase in spasticity grades was consistent with an increase in GMFM, while there was no correlation (p > 0.05) between spasticity grades and postural stability indices. Thus, changes in the spasticity grades were not consistent with changes in postural stability indices (Tables 2 and 3).

**Table 1:** Descriptive Statistics.

	N	Minimum	Maximum	Mean	Std. Deviation
PedsQL	45	60	85	70.6889	8.16818
GMFM	45	40.30	62.80	51.9333	6.94845
overall	45	2.50	4.80	3.4022	0.46048
ap	45	2.10	4.20	2.70202	0.40703
ml	45	1.20	3.70	2.5356	0.61132
Valid N (listwise)	45				

PedsQL = pediatric quality of life, GMFM = gross motor function measure, ap = anteroposterior, ml = mdiolateral.

**Table 2:** Correlations between spasticity grades, pedsQL, GMFM, and stability indices.

Spasticity grades	PedsQL	GMFM	Overall stability index	Antero-posterior stability index	Medio-lateral stability index
Correlation Coefficient (Spearman's) (ρ)	0.84	0.816	−0.092	0.177	−0.083
p-value	0.0001∗	0.0001∗	0.549	0.246	0.589

ρ: Spearman's correlation, p-value: probability value, GMFM: Gross Motor Function Measure, ∗significant <0.05.

**Table 3:** Correlations between pedsQL and GMFM stability indices.

PedsQL	GMFM	Overall stability index	Antero-posterior stability index	Medio-lateral stability index
Correlation Coefficient (Pearson's) (r)	0.931	0.429	0.401	0.003
p-value	0.0001∗	0.217	0.25	0.994

r: Pearson's correlation, p-value: probability value, GMFM: Gross Motor Function Measure, ∗significant <0.05.

The Pearson correlation between pedsQL and GMFM revealed a positive strong correlation (p < 0.05). This means that an increase in the Peds QL was consistent with an increase in GMFM, while there was no correlation (p > 0.05) between Peds QL and postural stability indices. This means that changes in the Peds QL were not consistent with changes in postural stability indices (Table 2).

## Discussion

The objective of this study was to determine whether there are associations between spasticity, motor function, postural stability, and life quality for children with cerebral palsy. The outcomes indicate a positive strong association between spasticity and health quality, as well as a strong significant association between spasticity and gross motor function, whereas no correlation was detected between spasticity and postural stability index. There was also a positive strong correlation between quality of life and gross motor function, but no association was identified between health quality and postural stability index.

The results of this study are reinforced by those of Gorter,^27^ who found that there is a strong positive association between spasticity and development of gross motor function in 18-month-olds.^27^ Muscular weakness, spasticity, selective motor control, and range of motion limitation directly compromise daily activities and gross motor tasks.^28^, ^29^, ^30^

A strong association between gross motor function and health quality percentage has also been identified.^31^^,^^32^ The results of the positive strong correction between gross motor and spasticity in our study are reinforced by previous studies that reported a positive relationship between motor function and spasticity.^30^^,^^33^^,^^34^

Gross motor function is considered to be a good index of the physical aspects of health life quality, but a poor index of the psychosocial element of HRQOL in these children.^35^

The results of the current study are confirmed by those of Gharaborghe et al.,^36^ who studied the association between gross motor function and health life quality in 60 children (4–12 year) diagnosed with CP who had been selected from different occupational therapy clinics. In this study, QoL was measured using CP-QoL and gross motor functions were tested with GMFM. The results revealed significant differences between gross motor function and QoL.^36^

In contrast, Dajpratham et al.^37^ reported that there is no relationship between spasticity and QOL in stroke patients.^37^ The differences between these findings and current finding could be attributed to culture differences, population type, and different measurement tools. In addition, Akodu et al.^38^ recognized that movement items and individual care of the HRQOL are strongly affected by spasticity in children with CP.^38^

Our findings are also aligned with those of other studies that have reported that children with CP that have gross motor function have advanced physical QOL^39^^,^^40^ Furthermore, Vanderslot et al.^41^ revealed that there is a positive association between gross motor function level and the physical quality aspects of HRQOL.^41^ By contrast, Dehno et al.^42^ indicated that motor function level may not modify quality of life.^42^

Gross motor functions were considered to be good predictors of the physical component of health life quality in children with CP.^35^ Finally the results of our study contradict those of Puspitasari et al.,^43^ who indicated that there was no significant relationship between gross motor function and QoL among children with CP after studying the relationship between gross motor function and quality of life among 31 children with CP aged 4–12 years.^43^

## Conclusion

The results of this study indicate that spasticity is the main cause of delayed gross motor function for children with spastic CP that influence life quality of these children with no impact on the postural stability in mild degrees of spasticity so, these results might be a suggestion to determine therapeutic interventions and priorities to plan the physical therapy program according to the main cause of disorder which leading to delay in multi-aspects of daily living activities.

## Recommendations

This study determined that spasticity has a positive effect on the motor function and health life quality of children with cerebral palsy; thus, we recommend that physiotherapists address spasticity as a first line of treatment to avoid delays in improving such children's ability to function normally and have a high quality of health.

## Source of funding

This research did not receive any specific grant from funding agencies in the public, commercial, or not-for-profit sectors.

## Conflict of interest

The author has no conflict of interest to declare

## Ethical approval

Approval was obtained from the ethics committee of the Faculty of Physical Therapy, Cairo University, Egypt (NO.P.T.REC|012|003020). Ethical approval date is 25/3/2020.
